# “If they were telling us to go, we will go”: factors associated with effective postnatal care coverage before and after discharge – a mixed-methods study in rural western Kenya

**DOI:** 10.1136/bmjgh-2024-016984

**Published:** 2026-03-13

**Authors:** Sein Kim, Beatrice Wamuti, Kennedy Opondo, Kevin Croke, Margaret Elizabeth Kruk

**Affiliations:** 1 Global Health and Population, Harvard T.H. Chan School of Public Health, Boston, Massachusetts, USA; 2 Kenyatta National Hospital, Nairobi, Nairobi County, Kenya; 3 Washington University School of Medicine in Saint Louis, St. Louis, Missouri, USA

**Keywords:** Health systems evaluation, Kenya, Child health, Maternal health, Prevention strategies

## Abstract

**Introduction:**

Few studies have explored the extent to which mother-newborn dyads have access to high-quality postnatal care (PNC) in low-income and middle-income countries. This study aims to measure effective (quality-adjusted) PNC coverage for newborns both before discharge and after discharge from the delivery facility, and for mothers before discharge, and to examine factors influencing access to high-quality PNC.

**Methods:**

We conducted an explanatory sequential mixed-method study in Kakamega County (Kenya) between January 2022 and November 2023. We collected quantitative data immediately after birth up to 60 days post partum and conducted in-depth interviews with mothers within the 60 days post partum. We present descriptive statistics of the effective PNC coverage cascade before and after discharge, with data analysed using multivariable logistic regression. Qualitative evidence was synthesised using thematic analysis.

**Results:**

Out of 611 births, 134 (22%) mothers and 468 (77%) newborns received effective PNC immediately after birth, respectively. By contrast, following discharge, only 171 (28%) newborns received effective PNC. Health systems factors, including antenatal care quality (OR 3.24, 95% CI 1.03 to 10.26), whether mothers received complete counselling (OR 2.20, 95% CI 1.29 to 3.76), and whether newborns received check-ups and immunisations before discharge (OR 2.21, 95% CI 1.01 to 4.79) were associated with effective PNC for newborns after discharge. Qualitative evidence from 36 interviews identified three main themes: quality of care for mother-newborn dyads before and after discharge; individual and interpersonal barriers and facilitators (including mothers’ perceptions of PNC, poverty and financial constraints, and information from social networks); and health systems-level barriers and facilitators (including communication and information on PNC, community outreach after delivery, and experience with quality healthcare influencing PNC use).

**Conclusions:**

PNC remains a weak point in the maternal newborn continuum of care, with low uptake and suboptimal quality. Efforts to enhance effective PNC, such as providing comprehensive counselling before discharge, are required across all levels of care.

**WHAT IS ALREADY KNOWN ON THIS TOPIC**

- Although immediate postnatal care (PNC) coverage has increased globally, PNC use has remained low compared with other components of maternal and newborn care, with individual-level barriers including financial constraints, distance to facilities and the low perceived need.

**WHAT THIS STUDY ADDS**

- Our findings highlight the importance of measuring effective PNC coverage separately for the predischarge and postdischarge periods to provide a more nuanced understanding of the continuum of care during postnatal period.
- Our study demonstrates a significant drop in both crude and effective PNC coverage, especially for newborns after discharge, indicating that many do not receive all necessary care components after leaving the delivery facility.
- Beyond individual-level barriers, how the health system communicates with mothers about PNC affects the continuum of care. Health systems factors, such as comprehensiveness of counselling, providers’ scheduling for the next PNC visit at the time of discharge, and the quality of antenatal care, influenced the use of postdischarge PNC for newborns.

**HOW THIS STUDY MIGHT AFFECT RESEARCH, PRACTICE OR POLICY**

- Provision of comprehensive and timely PNC needs to be re-emphasised across all levels of care.

## Introduction

 Maternal and newborn health is a key area of focus within Kenya’s health system, reflecting the global commitment to improving health outcomes for mothers and infants. Despite progress in recent years, Kenya continues to face significant challenges in reducing maternal and newborn mortality, with 530 maternal deaths per 100 000 births in 2020^1^ and stalled progress in reducing neonatal mortality. These deaths are largely avertable through provision of high-quality delivery and postnatal care (PNC), particularly during the first 42 days after birth, when opportunities to prevent adverse outcomes are the greatest.^2^ Such preventive measures include maternal and newborn physical assessment for complications, prevention of infections, and mental health and nutritional interventions provided within the first 24 hours, 3 days, 1–2 weeks and 4–6 weeks.^3^

Despite its importance, PNC in Kenya has received less attention than other elements of the maternal and newborn healthcare package. The Kenya Demographic Health Survey (DHS) in 2022 reported that 23% of mothers and 16% of newborns received no postnatal check-up within 2 days after birth, compared with just 2% of mothers who receive no antenatal care (ANC) at all (among women with a live birth or stillbirths in the preceding 2 years).^4^ There is also high geographical inequity in access to PNC across counties, with coverage rates ranging from 37% in Wajir County to 94% in Embu County.

The COVID-19 pandemic has further exacerbated gaps in access to PNC.^5^,^7^ In April 2020, Kenya introduced a policy document, “The Practical Guide for Continuity of Reproductive, Maternal, Newborn and Family Planning Care and Services in the Background of Covid-19 Pandemic”, which sought to minimise physical contact with health system personnel. Low-risk mothers undergoing normal delivery were guided to receive their first PNC at 6 weeks, while mothers who had undergone caesarean delivery (CS) were recommended to have visits at 2 and 6 weeks. High-risk mothers were to receive individualised care, although specific guidelines based on risk stratification were not indicated^8^ (online supplemental Table S1). One result of this policy is that while it remained in effect, most mothers with normal deliveries would have no immediate postnatal contact. While these changes were understandable during the height of the pandemic, this policy has remained active.

Previous observational and qualitative studies have highlighted additional barriers to PNC access for mothers and newborns in Kenya. At the individual level, rural residence, less than primary education, poverty and home delivery have been shown to be correlated with low PNC utilisation.^9^,^11^ Decision-making about care by other family members has also been shown to reduce PNC service utilisation.^12 13^ By contrast, mothers’ empowerment as principal decision-maker regarding health and knowledge of health danger signs were both associated with reduced delays in seeking PNC. At the health systems level, contextual factors such as nurse shortages due to strikes have also been shown to hinder PNC knowledge and uptake.^14^ A study in Kiambu and Nairobi counties also found that lower levels of trust in the health systems were associated with reduced PNC care seeking, especially after COVID-19.^15^

Going beyond PNC access, there is to date relatively limited research on quality of PNC throughout the 6-week postnatal period. Many existing studies overestimate coverage of PNC by measuring it as intrapartum care within 24 hours after childbirth,^16 17^ although comprehensive PNC, by definition, should occur throughout the postnatal period. Other studies have defined participants as having received PNC if they have received any check-up from a provider regardless of clinical content of care.^17 18^ For example, women may be considered as having received PNC even if they were not checked for vital signs during the visit. Relatedly, other studies have conflated postdelivery sick child visits with routine PNC visits. Moreover, research to date has primarily focused on demand-side determinants of receiving PNC, despite the multifaceted nature of this decision. Therefore, in this study, we seek to fill these gaps by providing a comprehensive assessment of barriers and facilitators to high-quality PNC access.

This study assessed crude and quality-adjusted PNC—the latter serving as a proxy for effective PNC to better reflect potential health gains.^19^ For mothers, we measured crude and effective PNC coverage only during the immediate postnatal period (before discharge). For newborns, we assessed both crude and effective PNC coverage before discharge and again during the early and late postnatal period (after discharge) in Kakamega, Kenya. We used both quantitative and qualitative data within 60 days post partum to contextualise the determinants influencing PNC-seeking behaviours in this setting.

## Methods

### Setting

Kakamega County, located in western Kenya, has a population of 1.9 million, a maternal mortality ratio of 316 per 100 000 live births, and neonatal mortality of 19 per 1000 live births in 2014.^20^ As of 2022, 73% of pregnant mothers received ANC four or more times, and 90% gave birth at facilities, a large increase from the 47% facility delivery rate recorded in 2014. During data collection, maternal and newborn care in Kakamega was free under the ‘Linda Mama’ programme, which covered delivery and PNC services for up to 1 year. Despite improved prenatal care and institutional delivery, access to PNC in Kakamega remains low, with only two-thirds of women receiving a physical check-up within the first 2 days after birth, below the national average of 73%.

### Study design

We conducted an explanatory sequential mixed-methods study (online supplemental Figure S1), in which the quantitative phase was followed by a qualitative phase. The qualitative phase was designed to explain and expand on the quantitative findings.^21^ Relying solely on quantitative data would have limited our ability to capture subjective beliefs and decision-making dynamics underlying PNC. Therefore, a mixed-methods approach was employed to leverage the strengths of both data sources, allowing for a comprehensive understanding of the factors influencing access to effective PNC.

First, we presented a quantitative analysis of effective PNC coverage, followed by a qualitative analysis using thematic analysis to explore barriers to and facilitators of access to postdischarge PNC. We integrated the quantitative and qualitative data during the design phase, where descriptive statistics from quantitative data informed recruitment decisions, such as age, experience of seeking PNC and the place of delivery. Using a contiguous narrative approach, we reported the findings from the two phases separately and discussed concordant and discordant findings.^22^

### Quantitative phase

#### Sample

Quantitative data were collected as part of an evaluation of a health system reform known as Service Delivery Redesign for Maternal and Newborn Health.^20^ The main data platform was known as the Kakamega Pregnancy Registry (KPR), which recruited pregnant women from primary and secondary health facilities across Kakamega County. The parent study intended to follow up approximately 3500 women longitudinally from enrolment at their first ANC visit, with interviews in their eighth month of pregnancy and in the month immediately post partum, with a final follow-up interview to 2 months after delivery. At the time of quantitative analysis, 611 out of 1068 participants had completed follow-up interviews and were within the 28 to 60 days postpartum window. These 611 women comprised our analytic sample. The longitudinal survey included questions regarding pregnancy histories, anthropometrics, biomarkers including systolic/diastolic blood pressure, access to and quality of ANC, experience with delivery services, and utilisation and content of PNC.

#### Outcomes of interest: crude and effective PNC coverage

We assessed crude and effective coverage on PNC as primary outcomes, in line with existing literature on this topic.^23^,^26^ Crude coverage was estimated as the proportion of women or newborns checked by providers among delivery cases at health facilities. Effective coverage of PNC was defined as the proportion of women or newborns in need of PNC services who had any contact with providers, and who received the recommended contents of care according to WHO and national guidelines (online supplemental Table S2),^24^ following a similar approach used in the literature.^19 27^ These measures were collected at specific time points, before and after discharge from delivery facility. We calculated effective coverage of PNC services as: effective PNC coverage=quality of PNC services × (utilisation of PNC services/population with need for PNC services).^23 26^ Detailed indicators are presented in online supplemental Table S3.

For mothers, PNC quality before discharge was measured by the proportion of women who received assistance with breastfeeding and were counselled on the following items: exclusive breastfeeding, cord care, thermal care, danger signs for both mothers and newborns, and the time and place for the next PNC visit. However, due to the nature of the question wording about PNC in our survey, we cannot measure postdischarge PNC for mothers during the 28-day follow-up. This limitation is further noted in the discussion section.

For newborns, PNC quality before discharge was measured as the proportion of vaccinated newborns among those who were checked while still present in the ward. PNC quality after discharge was assessed by the proportion of newborns who received five items (examination of the cord, measurement of temperature, weight and height, and vaccination) among those who had any PNC either at home or in facilities within 60 days after birth.

#### Quantitative data analysis

We present descriptive statistics graphically, to summarise crude and effective PNC coverage before and after discharge. Second, we constructed multivariable logistic regression models to investigate determinants of receiving any postdischarge PNC or effective PNC for newborns. Individual-level and health systems-level factors were selected based on existing literature and on correlations present in bivariate analysis. Individual-level factors included maternal age, education level, primigravida, household wealth, knowledge of free maternity services, length of travel to enrolment and delivery facilities, and whether mothers had caesarean deliveries. Health systems-related factors included place of delivery, number of ANC visits, quality of ANC, perceived satisfaction with delivery services, counselling, and newborn immunisation predischarge. We considered multiple births as a single delivery case. Standard errors were clustered at enrolment facility level to account for the sampling approach. Stata/MP V.18.0 and R V.4.3.2 were used for data analysis and presentation.

### Qualitative phase

#### Sample

We conducted in-depth interviews with 36 mothers living in Lugari and Malava subcounties using purposive sampling to capture variation. This sampling included mothers who had vaginal deliveries and those who experienced complications during delivery, many of whom underwent CS. Inclusion criteria were mothers aged over 15 years old, those who delivered at a subcounty hospital between November 2022 and mid-January 2023, and who were within the 60 days postpartum period. A main source of recruitment was a full list of the KPR enrollees. The study team recruited interviewees in two ways. First, community health volunteers (CHVs) contacted mothers and scheduled interviews in advance. Second, mothers were recruited at subcounty hospitals during their PNC visits.

#### Data collection

Qualitative data were collected from 30 January to 14 February 2023. The field research team consisted of five individuals (SK, KO, RK, HO and TRO). A semistructured interview guide, developed based on the research questions and existing literature, covered topics such as barriers and facilitators to accessing healthcare services after delivery, mothers’ user experience and perceptions of PNC. Interviews were conducted in a private place where mothers preferred, such as a private room at facilities, home or at markets near the interviewee’s house. IDIs were conducted in Kiswahili for approximately 45 min, and were audio-recorded, transcribed and translated into English by a separate experienced group of translators. Research assistants explained the consent form to participants, who signed the forms or provided their fingerprints to indicate consent. Interviewees received KES 200 (US$2) for their participation. Data collection continued until the saturation was reached; that is, the point at which no new information emerged from the interviews. For quality assurance, two translators worked on the 10% of the same audio file, and then the research team confirmed the consistency of translation. The field research team debriefed daily throughout the period in terms of sampling progress, emerging findings and any potential revision of interview guides.

#### Qualitative data analysis

We applied thematic analysis to generate themes about barriers and facilitators in access to PNC after delivery. Using a codebook approach, we first organised the analysis into deductive categories: contents of PNC, barriers and facilitators to accessing postdischarge PNC at individual, interpersonal and health-system levels, and mothers’ recommendations. Codes were generated reflectively, evolving throughout the analytic process until no additional codes provided new information.^28^ A total of 154 codes were created. Using Dedoose, SK coded, annotated and analysed the English transcripts, with 10% of the transcripts randomly selected to be independently coded by BW. The initial codebook was developed by SK and validated and refined by BW. Weekly meetings were held to confirm code agreement, resolve disagreements, revise the codebook and discuss emerging themes.

### Patient and public involvement

Patients and/or the public were not involved in the design, or conduct, or reporting, or dissemination plans of this research.

## Results

### Quantitative results

#### Sample characteristics

Table 1 presents descriptive statistics of our sample. Among 611 birth cases, the average maternal age was 25.7 years old. 192 (31%) of women were primigravida, 227 (37%) had anaemia measured at enrolment, 481 (79%) were married or de-facto living with a partner, 269 (44%) completed primary education and 214 (35%) belonged to the lowest household wealth group. The average time to reach ANC facilities was 36 min. For their most recent pregnancy, 485 (79%) mothers had at least four ANC visits, 256 (42%) of mothers delivered at primary healthcare facilities, 258 (42%) delivered at level 4 or higher public hospitals and 97 (16%) delivered at a private facility. 49 (8%) deliveries were through CS. On average, mothers stayed 1.4 days at facilities after delivery. Mothers reported that 280 (46%) newborns had symptoms of complications, including trouble breathing, vomiting, jaundice, infection or fever.

**Table 1:** Characteristics of respondents in 28–60 days of postnatal period

	n	Mean/proportion	SD
Individual level			
Mother’s age		25.74	5.97
Currently married or living with partner as if married	481	0.79	0.41
Maternal education level			
No education	100	0.16	0.37
Completed primary education	269	0.44	0.50
Completed secondary education or more	240	0.39	0.49
Wealth of household*			
Wealth quintile 1	214	0.35	0.48
Wealth quintile 2	40	0.07	0.25
Wealth quintile 3	117	0.19	0.39
Wealth quintile 4	131	0.21	0.41
Wealth quintile 5	109	0.18	0.38
Enrolled in NHIF or other private insurance	80	0.13	0.34
Knowledge of ‘Linda Mama’ programme	280	0.46	0.50
Length of travel to enrolment facility in minutes†		35.83	31.72
Length of travel to delivery facility in minutes		36.8	29.67
Risk factors			
Prior early neonatal death (0–28 days)	10	0.02	0.15
Gestational high blood pressure (after 20 weeks of pregnancy)	29	0.05	0.21
Gestational diabetes (>140 mg/dL) after 24 weeks of pregnancy	42	0.07	0.25
Anaemia (Hb <110 g/L)	227	0.37	0.48
Primigravida	192	0.31	0.46
Delivered by CS	49	0.08	0.27
Delivered multiples (twins or triplets)	7	0.01	0.11
Newborn had complication after birth‡	280	0.46	0.50
Health systems level			
Place of delivery			
Delivery hub§	225	0.37	0.48
Kakamega county general hospital	33	0.05	0.23
Public PHC facility in Kakamega	256	0.42	0.49
Private facility	97	0.16	0.37
Length of stay at maternity ward		1.44	1.58
At least 4 times of ANC	485	0.79	0.40
ANC quality index¶	388	0.64	0.19
Satisfaction on ANC services		3.22	0.51
Satisfaction on delivery procedure		3.73	0.54
Crude and effective coverage			
Crude coverage for mothers			
Before discharge, mothers checked by providers	475	0.78	0.42
Effective coverage for mothers			
Before discharge, effective PNC for mothers	134	0.22	0.41
Crude coverage for newborns			
Before discharge, newborns checked by providers	529	0.87	0.33
After discharge, newborn PNC visits (excluding sick visits)	324	0.53	0.50
Effective coverage for newborns			
Before discharge, newborn checked and vaccinated	468	0.77	0.42
After discharge, effective PNC for newborns	171	0.28	0.45
Observations	611		

* The wealth index was created using principal component analysis based on household assets: car, motorbike, television, electricity, pipe water, toilet, heating/cooling system, and roof and floor built with advanced materials.

† Travel time was self-reported regardless of the transportation mode. Missing observations were imputed as the mean travel time to enrolment facilities among individuals residing in the same ward or village.

‡ Newborn complications since discharge included trouble breathing, infection, trouble feeding, vomiting, diarrhoea, fever, cough, drowsiness or unresponsiveness, jaundice or failure to gain weight.

§ Delivery hub hospitals are level four hospitals newly equipped with resources that can provide caesarean sections, blood transfusions, focused care for sick newborns and other advanced services.

¶ The ANC quality index was calculated by averaging clinical and counselling items provided during each ANC visit. These items included weight, height, height of mother’s belly measurements, measurement of blood pressure, blood sample collection, syphilis and HIV testing, administration of tetanus toxoid vaccine and iron/iron folate supplements, counselling on healthy eating, place of delivery and scheduling for the next ANC visit.

ANC, antenatal care; CS, caesarean section; Hb, Hemoglobin ; NHIF, National Hospital Insurance Fund; PHC, primary healthcare; PNC, postnatal care.

#### Crude and effective coverage of PNC before and after discharge

**Figure 1:**
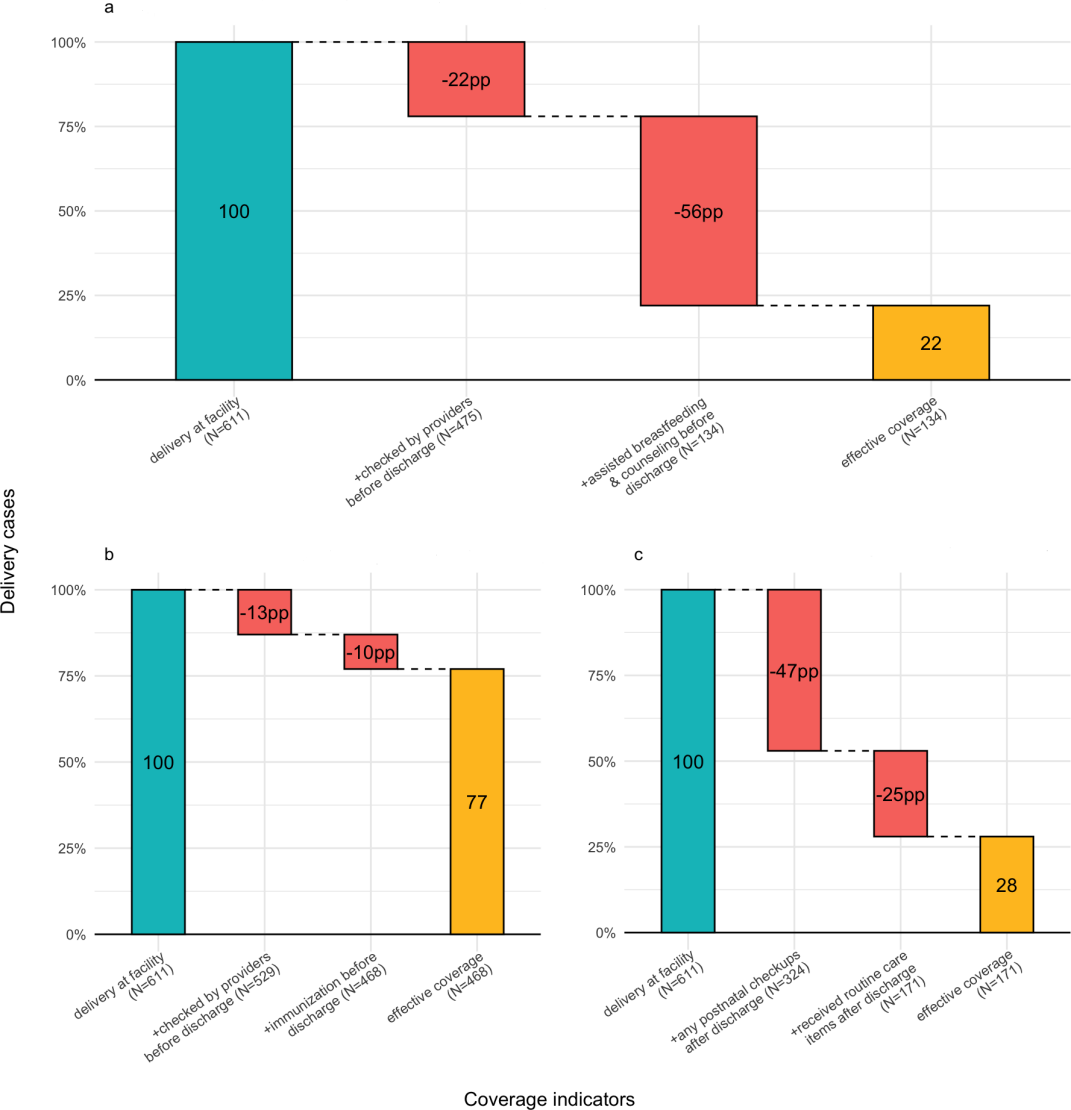
Effective coverage for mothers (before discharge) and newborns (before and after discharge). Notes: The denominator for calculating both crude and effective coverage of PNC was total delivery cases at facility in figure 1. (a) Presented effective PNC coverage cascade for mothers before discharge. For the numerator, crude coverage included a binary indicator of receiving a health check by providers before discharge. The numerator of effective coverage included indicators of assisted breastfeeding and eight counselling items: assisted breastfeeding, breastfeeding exclusively, care of the umbilical cord, the need to avoid chilling of the baby, danger signs for the baby’s health, danger signs for mothers’ health, when to come for a postnatal visit and where to go for a postnatal visit, conditional on receiving any health check by providers at the delivery facility. (b and c) displayed effective PNC coverage cascade for newborns before and after discharge. In (b), the numerator for crude coverage included a binary indicator of receiving a health check by providers before discharge. The numerator of effective coverage includes a binary indicator of receiving newborn vaccination before discharge conditional on receiving any health check by providers at the delivery facility. The numerator of crude coverage for (c) included a binary indicator of receiving a health check by providers. The numerator of effective coverage included indicators of five routine PNC items: examine the cord, measure the baby’s temperature, measure the baby’s weight, measure the baby’s length and vaccination conditional on receiving any health check by providers either at home or health facilities. PNC, postnatal care; PP, percentage point.

Crude PNC coverage before discharge, defined as any health checks by providers in the maternity ward, was 475 (78%) for mothers and 529 (87%) for newborns, out of 611 birth cases. Effective PNC coverage, assessing clinical actions provided according to national and WHO guidelines, was 134 (22%) for maternal care (figure 1a) and 468 (77%) for newborn care (figure 1b).

Figure 1c displays the crude and effective coverage of PNC after discharge for newborns. We excluded sick child visits whereby mothers visited facilities to treat specific illnesses like diarrhoea. In other words, we measured routine PNC for general check-ups. In 28–60 days after birth, 324 (53%) of newborns were examined by health providers either at home or at facilities. Effective coverage for newborns was 25 percentage points lower than crude coverage: only 171 (28%) received all components of routine PNC, such as examining the cord, and measuring temperature, weight and height.

### Association between postdischarge PNC for newborns and individual-level and health systems-level factors

Among individual-level factors, the completion of secondary education by mothers was associated with increased odds of accessing any follow-up visits (OR 1.84, 95% CI 1.19 to 2.82) or effective PNC for newborns (OR 1.74, 95% CI 1.08 to 2.79). Other factors, such as household wealth, knowing about the free maternity care programme, being a first-time mother and having a CS were positively, but not significantly, related to the outcomes (table 2).

**Table 2:** Association between the use of any or effective PNC after discharge and individual and health systems factors

	Any follow-up visits		Any PNC visits (excluding sick visits)		Effective PNC	
	OR	95% CIs	OR	95% CIs	OR	95% CIs
Individual level						
Mother’s age	0.99	(0.95 to 1.03)	1.00	(0.96 to 1.03)	1.00	(0.96 to 1.04)
Completed primary education (ref. no education)	1.37	(0.88 to 2.14)	1.27	(0.86 to 1.90)	1.46	(0.89 to 2.39)
Completed secondary education	1.84^**^	(1.19 to 2.82)	1.45	(0.93 to 2.26)	1.74^*^	(1.08 to 2.79)
Primigravida	1.18	(0.72 to 1.91)	0.94	(0.62 to 1.42)	1.08	(0.66 to 1.75)
Newborn experiencing any complication after delivery	1.15	(0.80 to 1.67)	–	–	–	–
Anaemia (Hb<110 g/L)	0.94	(0.68 to 1.30)	1.01	(0.71 to 1.42)	1.00	(0.65 to 1.55)
Wealth quintile						
Second (ref. lowest)	1.55	(0.77 to 3.13)	0.99	(0.49 to 2.03)	0.96	(0.39 to 2.34)
Middle	0.89	(0.52 to 1.53)	0.94	(0.61 to 1.46)	0.93	(0.59 to 1.47)
Fourth	0.96	(0.55 to 1.66)	0.99	(0.63 to 1.55)	0.98	(0.58 to 1.64)
Highest	1.22	(0.61 to 2.43)	1.2	(0.61 to 2.37)	1.28	(0.66 to 2.49)
Know Linda mama	0.84	(0.57 to 1.25)	1.13	(0.81 to 1.58)	1.1	(0.81 to 1.50)
Travel time (ref. ≤30 min)						
>30 min to enrolment facility	0.72	(0.49 to 1.06)	0.83	(0.60 to 1.15)	1.26	(0.90 to 1.76)
>30 min to delivery facility	1.34	(0.86 to 2.08)	1	(0.74 to 1.36)	1.19	(0.82 to 1.73)
Having c-section (ref. vaginal delivery)	0.98	(0.51 to 1.87)	1.28	(0.74 to 2.24)	1.88	(0.98 to 3.60)
Health systems level						
Antenatal care						
Numbers of ANC	0.96	(0.86 to 1.07)	0.93	(0.83 to 1.04)	0.91	(0.81 to 1.03)
ANC quality index	1.06	(0.38 to 2.95)	1.53	(0.60 to 3.90)	3.24^*^	(1.03 to 10.26)
Delivery care						
Place of delivery (ref. PHC)						
Delivery hubs	0.63^*^	(0.44 to 0.91)	0.70^*^	(0.52 to 0.95)	0.74	(0.50 to 1.09)
Private facility	0.72	(0.42 to 1.23)	0.66	(0.40 to 1.08)	0.60	(0.34 to 1.05)
Kakamega County General Hospital	0.98	(0.45 to 2.14)	1.16	(0.61 to 2.21)	0.99	(0.36 to 2.75)
Quality of delivery and immediate PNC						
Satisfied with delivery services	2.29^***^	(1.45 to 3.62)	2.69^***^	(1.77 to 4.07)	2.22^**^	(1.38 to 3.57)
Received complete counselling before discharge	2.13^***^	(1.36 to 3.33)	1.70^**^	(1.18 to 2.45)	2.20^**^	(1.29 to 3.76)
Newborn immunisation before discharge	1.48	(0.91 to 2.39)	1.21	(0.71 to 2.04)	2.21^*^	(1.01 to 4.79)
Observations	605		605		605	

Notes: Three outcomes were binary variables indicating whether newborns received any follow-up at facilities or at home, received PNC excluding sick visits or received effective PNC. Effective PNC was coded as 1 if the PNC visit was not for treating illnesses of the newborn and if the newborn received all five components: examination of the umbilical cord, measurement of temperature, weight and height, and child vaccination. Travel time to enrolment and delivery facility was a self-reported measure regardless of the transportation mode. Missing observations for travel time for enrolment and delivery facilities were imputed as the mean travel time to each facility among individuals residing in the same ward or village. The ANC quality index was calculated by averaging clinical and counselling items provided during each ANC visit. These items included weight, height, height of mother’s belly measurements, measurement of blood pressure, blood sample collection, syphilis and HIV testing, administration of tetanus toxoid vaccine and iron/iron folate supplements, counselling on healthy eating, place of delivery and scheduling for the next ANC visit. Satisfied with delivery variable was coded 1 if mothers rated the excellent service. 95% CI in brackets. SEs were clustered at enrolment facility level.

* p<0.05, **p<0.01, ***p<0.001.

ANC, antenatal care; Hb, Hemoglobin ; PHC, primary healthcare; PNC, postnatal care.

Health systems-level factors, such as the contents of immediate PNC before discharge, were significantly associated with receiving any PNC or effective PNC during the 28–60-day postnatal period. Mothers who received high-quality ANC had higher odds (OR 3.24, 95% CI 1.03 to 10.26) of obtaining effective PNC for their newborns. Also, mothers who rated delivery care as excellent had 2.69 times (95% CI 1.77 to 4.07) higher odds of seeking any routine PNC and 2.22 times (95% CI 1.38 to 3.57) higher odds of seeking effective PNC for their child. Mothers who received complete counselling before discharge had 1.70 times (95% CI 1.18 to 2.45) higher odds of seeking any routine PNC and 2.20 times (95% CI 1.29 to 3.76) higher odds of receiving effective PNC. Newborn immunisation before discharge was positively associated with effective PNC. The frequency of ANC or place of delivery was not consistently correlated with the outcomes.

### Qualitative results

We identified three main themes and six subthemes in the analysis. The main themes were: (1) quality of care for mother–newborn dyads before and after discharge; (2) individual and interpersonal barriers and facilitators, with subthemes including mothers’ perceptions of PNC, poverty and financial constraints, and information from social networks and (3) health system-level barriers and facilitators, with subthemes including communication and information on PNC, community outreach after delivery, and experience with quality healthcare influencing PNC use.

#### Sample characteristics

36 mothers participated in in-depth interviews (table 3). Among them, 34 (94%) were married, and 10 (28%) were primigravida. 10 mothers (28%) reported complications during labour and within 6 hours after delivery, with 27 (75%) having had a vaginal delivery and 9 (25%) undergoing CS. A majority of mothers and/or newborns received health services at health facilities. Over half of the visits were for newborn vaccination, followed by sick visits for issues like cord care or fever. Barriers and facilitators to accessing postdischarge PNC are explained more in detail in table 4.

**Table 3:** Characteristics of mothers participating in in-depth interviews (n=36)

Variable	n	Mean/proportion	SD
Age	36	26.31	5.95
Birth order for this baby	36	2.67	1.49
Experienced maternal complication	10	0.28	0.45
Mode of delivery			
Caesarean section	9	0.25	0.44
Normal	27	0.75	0.44
Had any visits to health facilities			
Not visited	2	0.06	0.23
Visited	30	0.83	0.38
Waiting to visit	4	0.11	0.32
Married	34	0.94	0.23
Newborns ever sick and/or weak	13	0.36	0.49
Reason for PNC visits within 8 weeks			
Not visited or mother’s check-up	9	0.25	0.44
Vaccination visits only	20	0.56	0.50
Sick visits	7	0.19	0.40

Notes: For maternal complications, mothers self-reported their experiences of any symptoms during labour or within 6 hours after delivery, including having seizures, losing consciousness, experiencing a higher-than-normal amount of bleeding, having a high fever, experiencing chills (shivering or shaking of the body, not due to feeling cold in a normal way), or having foul-smelling vaginal discharge.

PNC, postnatal care.

**Table 4:** Themes and selected quotes

Main theme	Subtheme	Code	Selected quotes
Quality of care for mother–newborn dyads before and after discharge		Quality of care before discharge	We were told to take our children to the clinic. Before we take them to the clinic, she taught those who were there how you are supposed to be breastfeeding (Lugari, ID 9, Age 24, with 1 child)
		Quality of care after discharge	R M: Has blood pressure been taken? R: No, they take blood pressure measurements while you are in the maternity and for the pregnant women alone. M: And for the postnatal mothers? R: They just attend to the baby’s alone unless you want to get or remove a family planning method. (Lugari, ID 17, Age 21, with 2 children)
			I got a method of family planning method. I wanted a five year implant but then I freaked out as I have never used it before and opted for the 3-month injection. However, the doctor counselled me and told me to consider another method in future as the 3-month one was not good if used for long. (Malava, ID 27, Age 24, with 3 children)
Facilitators for PNC			
Individual/interpersonal level	Mothers’ perception on PNC	Perceived PNC for baby as necessary	Because the child cannot explain whether they are sick so, that is the reason why you have to visit the hospital frequently. (Lugari, ID 25, Age 22, with 1 child)
		Perceived PNC for mother as necessary	M: And why do you think it’s important to attend post-natal care?R: It’s important because someone could be ailing unawares. So, the check-ups are good.M: Any other reason why it is important to attended post-natal care?R: The check-ups are good for both the mother and the babies as concerns their wellbeing and general health. (Lugari, ID 16, Age 24, with 1 child)
	Information from social networks	Friends and neighbours	Currently, I have neighbors who are of age, they provide me with information. In case the baby is sick, they advise me to take them to the hospital not anywhere else. (Malava, ID 3, Age 27, with 3 children)
Health systems-level	Communication and information on PNC	Appointment/referral letter	M: You were not told that you are supposed to bring the child for checkup from time to time? R: They told us to just take the child for the clinics on the written date (Malava, ID 5, Age 26, with 3 children)
	Availability of human resources after delivery	CHVs available	After I was discharged from hospital, he (community health volunteer) just visited me and ask me about the welfare of the child, whether when I was at the hospital if they treated me well? He asked some few questions, and I answered them but he always give me visits even today he was at my home. (Lugari, ID 12, Age 27, with 3 children)
	Experience on care quality influencing PNC use	Satisfied	They helped me. If it was in another place, maybe I would not have been assisted that night. Maybe I would have been told, “let us wait and see if the baby will turn. But, these ones assisted me hurriedly and though it was at night, they called the doctors at night and they came and assisted me. (Lugari, ID 14, Age 29, with 4 children)
		Expertise	It is always important to follow doctor’s instructions. When he tells you to come back for check-ups, it is better to do so as instructed because he knows why he is telling you to go back, and by doing this he may discover some problems which you didn’t even know while you were at home. (Malava, ID 2, Age 35, with 5 children)
Barriers for PNC			
Individual/interpersonal level	Mothers’ perception on PNC	Healthy/no demand/perceived normal	M: When they suffer from that [fever])what do you normally do?R: When they get such problems we assume that they are not sick and they stay at home until it is the day to go for clinic. (Lugari, ID 11, Age 37, with 6 children)I don’t see any need if I haven’t been sick, you know we have the notion that we must feel sick for us to go to the hospital. I cannot just go to have a general checkup (Lugari, ID 20, Age 31, with 3 children)
	Poverty and financial constraints to seek care	Poverty/expensive	M: Why were you not able to go early and waited till you saw pus oozing out? R: You might go and maybe you don’t have any money with you, and they ask you to pay some amount of money. (Lugari, ID 31, Age 24, with 4 children)
Health systems-level	Facility characteristics	Service delays	If it is here at 000, their services are at times very low generally; 000 is very low in service. If I was to leave the house and be there by 8, I would sit there till 10 without anyone bothering to serve me. It is possible to leave at noon after arriving at 8 AM (Lugari, ID 20, Age 31, with 3 children)
	Communication and information on PNC	Counsel not to come without challenge	You know we were not told anything, so we just know when the date reaches for the clinic, that’s when I take the baby and he gets injected and gets checked up. (Lugari, ID 29, Age 38, with 5 children) So, they (nurses) told me not to come back again for checkup but if I face any challenge I need to come back to the hospital. The remaining thing now is to take the baby to the clinic on the 13^th^ (Malava, ID 19, Age 34, with 3 children)
	Availability of human resources after delivery	No contact	M: And ever since you delivered have you ever visited you, a doctor or these village CHVs, have they ever come to see you and check on the baby?R: They have never come to check on the baby ever since I delivered. The leader is the one I usually see passing by and gives them vitamins. (Lugari, ID 32, Age 32, with 4 children)
	Experience on care quality influencing PNC use	Not satisfied	It was not good, and I did not see any need of coming. I expected to be given drugs cleaning the CS Scar and if there were abnormal discharge what I need to do but the doctor said that was normal. After I asked him about the baby he did not care and told me to go to the bab’’s clinic and that is where I will get help. (Malava, ID 19, Age 34, with 3 children)
Recommendations	Recommendation— mother	Additional clinical services	I think that they should give people an appointment to come back at least after 2 weeks. You know if they write for you an appointment, you will know that you are supposed to go back but if it is not written, you cannot come back (Lugari, ID 14, Age 29, with 4 children)
		Home visits	If they visit us, that will be good because if you will say you are planning to go to the hospital, you will keep postponing because of commitments. But if they visit, it will be easier. (Malava, ID 3, Age 27, with 3 children)
		Joint visits	The doctors need to take care of the mothers when they come for checkup and if there is any medication, they need to offer that. They don’t need to wait for six weeks to check on the baby but when the mothers come along with the babies for checkup, they need to ask how the baby is faring. When they just tell you that you are okay and just go back home is not enough. (Malava, ID 19, Age 34, with 3 children)

CHVs, community health volunteers; PNC, postnatal care.

### Quality of care for mother-newborn dyads before and after discharge

Right after delivery, mothers mentioned that providers rotated regularly to monitor blood pressure, bleeding, progress of breastfeeding and their general condition, and to advise on family planning. A comprehensive physical examination was uncommon at the time of discharge. At discharge, most mothers received a written schedule in the maternity book for newborn immunisation after 6 weeks without clear instruction on routine PNC for themselves or their newborns. The common content of counselling included advice about how to breastfeed newborns and the need for family planning but did not typically include advice on danger signs or how to seek help for mothers’ health.

During the first 6-week PNC visit, the care focus was primarily on newborns; however, comprehensive growth evaluation or nutritional counselling for infants was rare. Services such as checking the baby’s navel area, eyes and taking temperature were seldom mentioned. For maternal care, mothers reported receiving family planning counselling and/or services on the same day as the child’s vaccination if they had not received these services immediately after delivery. However, mothers reported that providers rarely checked maternal conditions like blood pressure, signs of complications, pain or mental health. For example, one mother explained that PNC services were not attentive to mothers:

Moderator (M): Has your blood pressure been taken?

Respondent (R): No, they take blood pressure measurements while you are in the maternity ward and only for pregnant women.

M: And for postnatal mothers?

R: They just attend to the babies unless you want to get or remove a family planning method. (Lugari, ID 17, Age 21, with two children)

Instead, common counselling topics focused on exclusive breastfeeding, vitamin intake, healthy eating and keeping the baby warm. Some mothers sought wound care or dealt with complications earlier than scheduled vaccination, but they left with medication only.

### Individual-level and interpersonal-level facilitators and barriers

#### Mothers’ perception of PNC

Mothers’ perceptions of the need for and usefulness of PNC varied and influenced their demand for care. While over 70% of interviewed mothers recognised the importance of PNC, many prioritised it for newborns over themselves. This perception was shaped by information received during ANC and immediately after childbirth, as well as common practices and norms in communities.

Mothers underscored that receiving PNC is crucial for identifying unforeseen medical issues, for preventing severe illnesses and for promoting general well-being of themselves and their newborns. First-time mothers and those with small newborns stressed that regular PNC could be necessary to monitor the baby’s health and to receive guidance on breastfeeding. In addition, some mothers expressed that PNC was essential for their own health. Mothers who experienced complications such as hypertensive disorders emphasised the importance of general check-ups to make sure that their body had fully recovered.

On the other hand, some women believed that PNC was not routine care, considering it necessary only in the event of complications or scheduled visits for specific purposes like child immunisation. For example, one mother said:

I don’t see any need if I haven’t been sick, you know we have the notion that we must feel sick for us to go to the hospital. I cannot just go to have a general checkup (Lugari, ID 20, Age 31, with three children)

When mothers and their newborns were both healthy, they expressed little demand for PNC. Some mothers even feared visiting facilities before scheduled dates without clinical reasons, leading mothers to have a ‘wait and see’ approach by allowing illnesses to develop further until the first PNC appointment after 6 weeks.

#### Poverty and financial constraints

Mothers often struggled to afford transportation and counselling costs, deterring timely PNC attendance. Despite ostensibly free maternity services, in many cases, facility deliveries already caused mothers to incur unexpected expenses for medical supplies. Fear of similar additional costs discouraged mothers from seeking PNC. For example, one mother with a CS avoided necessary wound care due to the concerns about being asked to pay for services. Additionally, competing financial demands, such as providing meals and essentials for families, further limited the household healthcare budget. As breadwinners, working mothers faced increased burdens balancing between working and seeking healthcare on time and were often forced to choose to work.

Nevertheless, they tended to visit facilities for child vaccination, prioritising newborn health. Scheduled vaccinations at 6 weeks seemed to provide caregivers with enough time to prepare finances for PNC. The issue of healthcare cost typically arose when mothers needed care for themselves, resorting to purchasing medicines at pharmacies or relying on traditional remedies.

#### Information from social networks

Mothers’ social networks played dual roles as both facilitators of and barriers to seeking PNC, because mothers received advice from various sources including grandmothers, mothers-in-law, friends and neighbours. Shared decision-making on use of care and appropriate places for PNC involved inputs from these people. For instance, experienced neighbours helped mothers assess whether a newborn’s symptoms required immediate medical attention, particularly supporting young and first-time mothers who lacked other sources of guidance.

Conversely, mothers’ social network at times prevented PNC-seeking through misinformation. The concept of routine PNC was rarely shared at community level. For example, in some cases, neighbours advised mothers to follow traditional practices in place of seeking medical attention when a newborn had prolonged fever. In other cases, illnesses were mistaken as a normal part of child growth. As a result, some mothers did not go to facilities, believing these symptoms to be part of a newborn’s natural adjustment to the new environment.

### Health systems-level barriers and facilitators

#### Communication and information on PNC

Short lengths of stay in health facilities, often less than 24 hours, provided insufficient time for mother-newborn dyads to receive comprehensive PNC. In this case, the crucial window for informing mothers was missed given the much higher chance for interactions with health workers during this time compared with postdischarge. Reasons for early discharge included lack of space, discomfort, unhygienic conditions or perceived lack of attention from providers. Only a few mothers recalled communication challenges with providers, stemming from rudeness, harassment or neglect once delivery was done.

Even when time allowed for PNC-related communication before discharge, over half of mothers reported being rarely counselled on integrated care of the mother-newborn dyads, as counselling primarily focused on newborn care. Mothers recalled little discussion about their physical and mental well-being, nutrition and discussion of various family planning methods was also limited, while emphasis was instead placed on a balanced diet for breastfeeding and exclusive breastfeeding. A few mothers received information on newborn danger signs, such as refusal to breastfeed or fever, and were advised to visit facilities on those occasions.

Furthermore, mothers mentioned that providers often undervalued the importance of routine PNC, advising them to return only after 6 weeks for immunisation. One interviewee recalled that:

You know, we were not told anything, so we just know when the date reaches for the clinic, that’s when I take the baby and he gets injected and gets checked up. (Lugari, ID 29, Age 38, with five children)

Exceptions were made for mothers who delivered through CS, as they were told to return for a check-up after 2 weeks. This guidance influenced mothers to believe that PNC visits were only necessary for scheduled appointments or in the event of medical problems. The clinic date recorded in their maternity books served as a reminder for PNC and encouraged mothers to plan their visit accordingly. However, some mothers reported being explicitly told not to return to the facility unless they encountered health challenges, which further diminished their demand for PNC. In rare cases, mothers were not even scheduled for child vaccinations.

#### Community outreach after delivery

Following discharge, mother-newborn dyads often became disconnected from the health system. Many mothers lost regular contact with community health volunteers after childbirth; only 5 (14%) met CHVs in the postnatal period. While some active CHVs visited homes to teach newborn care and encouraged follow-up visits, most provided less support than during pregnancy. Mothers expressed concern about this loss of contact with CHVs, noting that continued support could help identify complications and address questions promptly. Ongoing CHVs monitoring was often reported by mothers with complications or those who had undergone a CS.

#### Experience with quality healthcare influencing PNC use

We found that satisfaction with care—reflected in mothers who reported experiencing excellent and respectful care during delivery—fostered patient-provider trust, encouraging mothers to adhere to medical advice, including seeking PNC on time. While mothers generally expressed high satisfaction with the delivery service as they mostly had safe delivery and healthy newborns, they also valued other aspects of care. Mothers were largely content with providers’ frequent monitoring, opportunities to ask questions and administration of tests, and respectful attitudes, such as treating mothers with dignity and going the extra mile to explain detailed information. Mothers who had these positive experiences assessed that providers possessed superior knowledge and offered effective solutions, which motivated them not to miss PNC as instructed.

Moreover, mother’s initial experiences with PNC contributed to decreased demand for subsequent PNC. Half of the interviewees expressed greater dissatisfaction with their first PNC visits compared with delivery services. Mothers cited issues such as insufficient clinical items and counselling, lack of medicines and health professionals, and a lack of attention at the maternal and child health department for PNC visitors, resulting in long queues. Also, mothers were more inclined to bypass nearby primary health facilities (perceived as lower quality), which increased time and cost associated with PNC, further hindering timely use of PNC services.

## Discussion

This study provides a comprehensive understanding of the multilevel factors associated with access to effective PNC. Quantitative evidence found that clinical quality of PNC lagged coverage, with a substantial gap between crude and effective coverage measured for newborns both before and after discharge, and for mothers before discharge. Health system factors, such as predischarge care content, were associated with this substandard PNC coverage, more than with individual-level factors. Qualitative findings generally confirmed the quantitative results, while offering new insights into mothers’ beliefs about the usefulness of PNC and the role of social networks in making decisions to either seek or forego PNC—an aspect not explored in the quantitative phase (see online supplemental figure S2 for the summary).

Both quantitative and qualitative evidence demonstrated a coverage gap in PNC, highlighting the need for greater attention to provision of high-quality PNC, particularly for mothers. Our quantitative results aligned with the 2022 Kenya DHS, which found that only 25% of mothers and 54% of newborns received quality PNC within 2 days after delivery.^4^ This consistent gap between mother and newborns consistently shed light on the neglect of maternal care before discharge, as observed in our study. Moreover, we found suboptimal effective PNC coverage for newborns after discharge, a gap that existing survey tools do not capture. The subsequent decrease in the uptake and quality of PNC indicated disengagement of mother-newborn dyads from the health system, compounded by limited attention by CHVs or traditional birth attendants, similar to what has also been observed in the South-Eastern state in Nigeria.^29^

Beyond examining gaps, qualitative findings showed that mothers’ decision to seek PNC on time was often influenced by their perceptions about the value of PNC and was shaped by their care experiences and interaction within their social circles. This result was consistent with a qualitative synthesis of 59 studies, which indicated that social norms prioritising PNC solely for infants, a lack of trust in the health systems and mothers feeling overlooked in their own care needs contributed to decreased PNC uptake.^13^ Similarly, a study across 12 LMICs found that low PNC use was affected by family members, who often underestimated its necessity and valued traditional postnatal practices.^12^

The overall findings supported a positive relationship between high-quality care during the immediate postnatal period and demand for PNC in the later postnatal period. Other studies have supported this link between quality of care and the continuum of care.^18 30 31^ For example, in western Ethiopia, Kasaye *et al* found that women who experienced abuse and discrimination during maternity care were less likely to complete the continuum of care.^31^

Specifically, interaction with health providers was critical to help mothers understand the need for comprehensive PNC, and accordingly for its utilisation.^13^ Providers who failed to emphasise the rationale for routine PNC led mothers to understand PNC as only needed in case of illness. Undervaluing of PNC has been observed in other countries, including Nigeria,^29^ Uganda^32^ and Zambia.^33^ In addition, delays in PNC related to COVID-19 were consistent with the broader negative impact of the pandemic on access to routine primary care, as reported in other LMICs.^734^,^36^

Although mothers’ socioeconomic factors, such as poverty, influenced access to high-quality PNC in the qualitative findings, this result was not confirmed in the quantitative findings. Rising food insecurity in the region^37^ and bypassing patterns to higher-level delivery facilities^38^ increased healthcare costs, further delaying PNC visits. This discordance may be explained by the focus of the quantitative phase on newborn PNC, where financial constraints were less critical, whereas the qualitative phase explored broader constraints, including the burden of PNC visits for mothers. Additionally, the positive association between maternal education and effective PNC observed in the quantitative results, but not explored in the qualitative phase, suggested that educated mothers might seek high-quality facilities or possess higher health literacy to seek more preventive care, such as child vaccination.^39^

Several policies and practices are recommended to improve effective PNC coverage in this setting. First, returning to pre-COVID-19 guidelines is necessary. This transition will shorten PNC schedules from 6 weeks to at least 2 weeks after birth, encouraging clients to use PNC on time who might not otherwise seek care without designated appointments. Moreover, greater provider attention and improved practices in delivering comprehensive PNC throughout the postnatal period are needed. Delivery at higher level facilities, presumably with better readiness, was not associated with seeking of PNC for newborns, suggesting inadequate instructions and insufficient service provision across all levels of care. Third, facilities can consider offering integrated PNC for mother–newborn dyads to reduce costs and enhance access. Lastly, revitalising the community health systems can help detect risks, facilitate referrals especially for the rural poor^6 40 41^ and improve maternal mental health during the transition to motherhood.^42^

This study is among the few to measure effective PNC coverage accounting for both quality and timing, using longitudinal data. Our findings provide a more comprehensive picture of how mothers navigate health systems to access PNC services. Our insights highlight that the quality of an individual’s most recent health system experience can significantly influence perceptions and health-seeking patterns throughout the postnatal period.

This study has several limitations. Our analytic samples were confined to mothers who delivered at facilities, and in a specific region of western Kenya. These restrictions reduce the generalisability of the findings to home births or other regions of Kenya. Second, our measures for assessing the quality of PNC before and after discharge were constrained by data, resulting in a less-than-complete view of PNC quality. For example, we could not capture effective PNC coverage for mothers after discharge due to the survey question design, which did not allow us to ascertain the timing of PNC. Additionally, using child immunisation as a proxy for quality of predischarge PNC for newborns can be less comprehensive than other studies which have incorporated other items such as immediate skin-to-skin care and clean umbilical cord care.^18 43^ Further study is needed to develop consistent and comprehensive measures of PNC for mother-newborn dyads to address remaining gaps. Lastly, reliance on self-reported data on PNC might introduce recall bias.

## Conclusions

This study confirms that PNC is a weak point in the maternal newborn continuum of care. To prevent adverse health outcomes associated with missed or suboptimal PNC for mothers and newborns, the provision of comprehensive and timely PNC needs to be re-emphasised across all levels of care.

## Supplementary material

10.1136/bmjgh-2024-016984 online supplemental file 1

## Data Availability

Data are available on reasonable request.
