# Supplementary material for: “If they were telling us to go, we will go”: factors associated with effective postnatal care coverage before and after discharge – a mixed-methods study in rural western Kenya
Source: BMJ Glob Health. 2026 Mar 13;11(3):e016984. doi: 10.1136/bmjgh-2024-016984 (PMC12993366; doi:10.1136/bmjgh-2024-016984)
Supplement: online supplemental file 1 [file bmjgh-11-3-s001.docx]

**“If they were telling us to go, we will go”: Factors associated with effective postnatal care coverage before and after discharge– a mixed-methods study in rural Western Kenya**

**Supplementary materials**

[Tables]

Table S1. A guideline for postnatal care (PNC) service before and after Covid-19 in Kenya

Table S2. Comparison between Kenya guidelines and WHO recommendation on postnatal care (2022)

Table S3. Quality indicators for postnatal care for mothers and newborns

[Figures]

Figure S1. Mixed-methods study design

Figure S2. Summary of qualitative analysis

Table S1. A guideline for postnatal care (PNC) service before and after Covid-19 in Kenya

|  | Before Covid-19 (Pre-2020) | After Covid-19 (Post 2020) |
| --- | --- | --- |
| Mother |  |  |
| timing of PNC visits | within 24 hours after birth |  |
|  | 1-2 weeks | after 2 weeks for low-risk women with caesarean section (CS) |
|  | 4-6 weeks | after 6 weeks for low-risk women with vaginal delivery |
|  | 4-6 months |  |
|  |  |  |
| frequency | at least four PNC checks within 42 days for all delivery cases | no PNC checks within 42 days for vaginal deliveries |
|  |  | one PNC check within 42 days for CS |
| Newborn |  |  |
| child immunization & growth monitoring | every 4 weeks | at 6, 10, 14 weeks and 6 months |

Source: Policy guidelines for postnatal care (PNC) in Kenya were based on *Healthy Mothers and Newborns: Guidelines for Postnatal Care* before Covid-19, and on “*The Practical Guide for Continuity of Reproductive, Maternal, Newborn and Family Planning Care and Services in the Context of the COVID-19 Pandemic*” after Covid-19.

Table S2. Comparison between Kenya guidelines and WHO recommendations, and quantitatively measured items

|  | **Healthy mothers and newborns: Guidelines for postnatal care in Kenya (2018)** | **WHO recommendation on PNC (2022)** |  |
| --- | --- | --- | --- |
| Target | Content of PNC | Content of PNC | Measured items in this study |
| mothers | Regular assessment of the women (pulse rate, blood pressure, excessive vaginal bleeding, uterine contraction, temperature, respiratory rates, signs of infection, leakage of urine) | Physiological assessment of the women | Yes, partially |
|  | Assisted, early initiation of breastfeeding | Assisted breastfeeding | Yes, partially (counseling only) |
|  | Information on nutrition, exclusive breastfeeding, hygiene, family planning, and birth spacing  Advice on reporting any health concerns to a health care provider: signs and symptoms of postpartum hemorrhage, pre-eclampsia/eclampsia, infection, or thromboembolism. | Information provision, educational interventions and counselling are recommended to prepare women, parents and caregivers for discharge from the health facility after birth to improve maternal and newborn health outcomes, and to facilitate the transition to the home. | Yes, partially |
|  | Schedule for postnatal care contacts (a minimum of four postnatal care contact) | Schedule for postnatal care contacts (a minimum of four postnatal care contact) | Yes |
|  | Check for calf tenderness | Local cooling and oral analgesia for perineal pain relief | No |
|  |  | Prevention of postpartum constipation | No |
|  | Assessment for mental or psychological wellness | Screening and prevention for postpartum depression and anxiety | No |
|  |  | Physical activity and sedentary behavior | No |
|  | Care for healthy women and newborns in the health facility is recommended for at least 24 hours after vaginal birth. | Care for healthy women and newborns in the health facility is recommended for at least 24 hours after vaginal birth. | No |
|  | Home visits for postnatal care contacts | Home visits for postnatal care contacts | No |
|  | Postpartum contraception | Postpartum contraception | No |
|  | Iron and folic acid supplementation | Iron and folic acid supplementation (context-specific) | No |
|  | Encouragement of male partner to support the women |  | No |
|  | All HIV-infected women should be offered ART as per the Kenya ART guidelines | Catch-up postpartum HIV testing is needed for women of HIV-negative or unknown status who missed testing | No |
| newborns | Full clinical examination within an hour after birth and before discharge | Full clinical examination within an hour after birth and before discharge | Yes, partially |
|  | Assessment of the newborn for danger signs: not feeding well; history of convulsions; fast breathing (breathing rate > 60 per minute); severe chest in-drawing; no spontaneous movement; fever (temperature > 37.5 °C); low body temperature (temperature < 35.5 °C); any jaundice in first 24 hours after birth, or yellow palms and soles at any age. | Assessment of the newborn for danger signs: not feeding well; history of convulsions; fast breathing (breathing rate > 60 per minute); severe chest in-drawing; no spontaneous movement; fever (temperature > 37.5 °C); low body temperature (temperature < 35.5 °C); any jaundice in first 24 hours after birth, or yellow palms and soles at any age. | Yes, partially (counseling only) |
|  | Cord care | Clean, dry umbilical cord care | Yes |
|  | Immunization for the prevention of infections | Immunization for the prevention of infections | Yes |
|  | 1% tetracycline eye ointment should be administered |  | No |
|  | Vitamin K should be administered |  | No |
|  | Immediate skin-to-skin care | Immediate skin-to-skin care | No |
|  | Universal screening for abnormalities of the eye | Universal screening for abnormalities of the eye | No |
|  |  | Universal screening for hearing impairment | No |
|  |  | Universal screening for neonatal hyperbilirubinaemia | No |
|  | Timing of first bath to healthy newborn to be delayed for at least 24 hours after birth. | Timing of first bath to healthy newborn to be delayed for at least 24 hours after birth. | No |
|  |  | Sleeping position for the prevention of sudden infant death syndrome | No |
|  |  | Whole-body massage | No |
|  |  | Early childhood development | No |

Note: Items marked as "Yes, partially" in the measured items column indicate that they are related to specific components of postnatal care checks. However, the full components were not practiced and/or were instead discussed as part of counseling. For example, the physiological assessment of women was counted as "Yes" or "No," without assessing each specific component of the physiological assessment, such as pulse rate, blood pressure, or temperature.

Table S3. Quality indicators for postnatal care for mothers and newborns

| Target | Indicators | nominator | denominator |
| --- | --- | --- | --- |
| Care during immediate postnatal period at maternity ward/before discharge | | | |
| mothers | crude coverage | number of mothers examined by providers at maternity ward | number of delivery cases at facility |
|  | effective coverage (8 items) | number of mothers examined by providers at maternity ward, received breastfeeding assistance from providers and who received all 7 counseling items  (1) breastfeeding exclusively  (2) care of the umbilical cord  (3) need to avoid chilling of baby  (4) danger signs for baby’s health  (5) danger signs for mothers’ health  (6) when to come for a postnatal visit  (7) where to go for a postnatal visit | number of delivery cases at facility |
| newborns | crude coverage | number of newborns examined by providers at maternity ward | number of delivery cases at facility |
|  | effective coverage (1 item) | number of newborns received a vaccination | number of delivery cases at facility |
| Care during postnatal period after birth | | | |
| newborns | crude coverage | number of newborns checked health by providers at home or at health facility | number of delivery cases at facility |
|  | effective coverage (5 items) | number of newborns checked health by providers at home or at health facility and  received 5 postnatal care items  (1) examine the cord  (2) measure the baby’s temperature  (3) measure the baby’s weight  (4) measure the baby’s length  (5) give vaccination | number of delivery cases at facility |

Notes: PNC coverage before discharge was assessed using the survey conducted seven days after delivery, while PNC coverage after discharge was based on the 28-day follow-up survey. For mothers, the survey question was: “After the delivery, did any health care provider or traditional birth attendant check on your health either at home or at a health facility?” Because this question did not distinguish whether the check occurred before or after discharge, we were only able to measure PNC coverage before discharge for mothers. For newborns, however, PNC coverage was measured both before and after discharge.

Figure S1. Mixed-methods study design

Note: We adopted an explanatory sequential design in which we quantitatively measured the crude and effective coverage of postnatal care (PNC) for mothers and newborns through secondary data analysis. At the time of the initial quantitative analysis in early 2023, 367 mothers had been followed up and completed the endline survey conducted after 60 days delivery. Of these, 267 women were in the 28-60 days postpartum period. Subsequently, we used qualitative data to explain how individual-, interpersonal-, and health-systems level factors influenced the use of PNC before and after discharge. At the time of the quantitative analysis in the second phase, 1,068 women completed the endline survey and 611 women were in 28-60 days postpartum period. This final group of 611 women constituted our analytic sample of quantitative phase. This study triangulated both data sources to understand the barriers and facilitators for utilizing high-quality PNC and synthesized the results in the discussion section.

Figure S2. Summary of qualitative analysis

Notes: Mother-newborn dyads experience barriers and facilitators at individual-, interpersonal-, and health-systems levels during different postnatal period, both before and after discharge. Overall, the majority of barriers and facilitators were identified at the health systems-level, while perception on postnatal care (PNC) and health status influenced mothers’ decisions to seek PNC.
